# Tumor Cell Distinguishable Nanomedicine Integrating Chemotherapeutic Sensitization and Protection

**DOI:** 10.3389/fbioe.2021.773021

**Published:** 2021-11-08

**Authors:** Sen Liu, Can Shen, Cheng Qian, Jianquan Wang, Zhongmei Yang, Yanchun Wei, Li Quan, Changjiang Pan, Yong Hu, Wei Ye

**Affiliations:** ^1^ Jiangsu Provincial Engineering Research Center for Biomedical Materials and Advanced Medical Devices, Faculty of Mechanical and Material Engineering, Huaiyin Institute of Technology, Huaian, China; ^2^ Institute of Materials Engineering, Collaborative Innovation Center of Chemistry for Life Sciences, College of Engineering and Applied Sciences, Nanjing University, Nanjing, China

**Keywords:** chemotherapy, NIR-controlled release, cell distinguishing, chemotherapeutic sensitization, chemotherapeutic protection

## Abstract

Theoretically, with a high enough drug dosage, cancer cells could be eliminated. However, the dosages that can be administered are limited by the therapeutic efficacy and side effects of the given drug. Herein, a nanomedicine integrating chemotherapeutic sensitization and protection was developed to relieve the limitation of administration dosage and to improve the efficacy of chemotherapy. The nanomedicine was endowed with the function of synergistically controlled release of CO and drugs under near-infrared (NIR) light irradiation. CO photo-induced release system (COPIRS) was synthesized by constructing an electron excitation–electron transfer group–electron-induced CO release structure and was used as the hydrophobic part, and then hydrophilic polymer (polyethylene glycol; PEG) was introduced by a thermal-responsive groups (DA group), forming a near-infrared-induced burst-release nanocarrier. *In vitro* and *in vivo* experiments showed that the nanomedicine can distinguish between tumor and normal cells and regulates the resistance of these different cells through the controlled release of carbonic oxide (CO), simultaneously enhancing the efficacy of chemotherapy drugs on tumor cells and chemotherapeutic protection on normal cells. This strategy could solve the current limitations on dosages due to toxicity and provide a solution for tumor cure by chemotherapy.

## Introduction

Chemotherapy is a widely used cancer treatment that could theoretically cure tumors provided that a sufficiently high drug dose is administered (Fung and Travis, 2018). However, the dose-dependent toxicity of chemotherapy drugs severely limits the maximum-tolerated dose (MTD), leading to an inability for the drug dose to reach cure and greatly restricting the clinical effect of chemotherapy (Constantinec et al., 2019). Chemotherapeutic sensitization (CS) aims to enhance the therapeutic efficiency of drugs by enabling cure at the MTD (Jain and Stylianopoulos, 2010; Cheng et al., 2015; Gonzalez et al., 2018; Vermaas et al., 2018; Liu et al., 2019), whereas chemotherapeutic protection (CP) is designed to protect normal cells from the toxicity of drugs in order to increase the administration dosage (Zhou and Bartek, 2004; Henry et al., 2018). However, to date, tumor cure has not yet been achieved, mainly due to the complex physiological environment and heterogeneity of tumors *in vivo* weakening the effect of CS or CP, failing to achieve cure at the MTD (Vines et al., 2019). The combination of CS and CP could not only reduce the dose required for cure by enhancing the efficacy of drugs on tumors but could also increase the MTD by protecting normal cells from the toxicity of drugs, which might be an effective strategy to break through the limits of MTD on the effect of chemotherapy.

However, as the mechanisms of CS and CP were aimed to alter the inhibition effect of drugs on cells in two opposite directions, it is difficult to combine them in a unified therapeutic system and, to the best of our knowledge, there has been no report on the combination of CS and CP in a unified therapeutic system.

In recent years, carbonic oxide (CO) has been proven to differentiate between tumor and normal cells, when used in combination with chemotherapy drugs (Zheng et al., 2017; Zhang et al., 2019). CO could simultaneously enhance the efficacy of chemotherapy drugs on tumor cells through CS and improve the drug tolerance dose of normal cells through CP (Wegiel et al., 2013), acting as a factor to distinguish tumor cells from normal cells. However, reactive oxygen species in the process of CS have a short half-life and their effect is therefore time dependent (Deng et al., 2020). And the discrete administration of CO (or its controlled-release materials; Ling et al., 2018) and chemotherapy drugs is difficult to ensure the effect of CO on CS and CP. Thus, ensuring the spatial and temporal consistency between the delivery and release of CO and chemotherapeutic drugs is a key problem that has not been reported in the application of CO, and would maximize the role of CO and significantly improve its CS and CP effects, thus further improving the effect of tumor chemotherapy.

**FIGURE 1:**
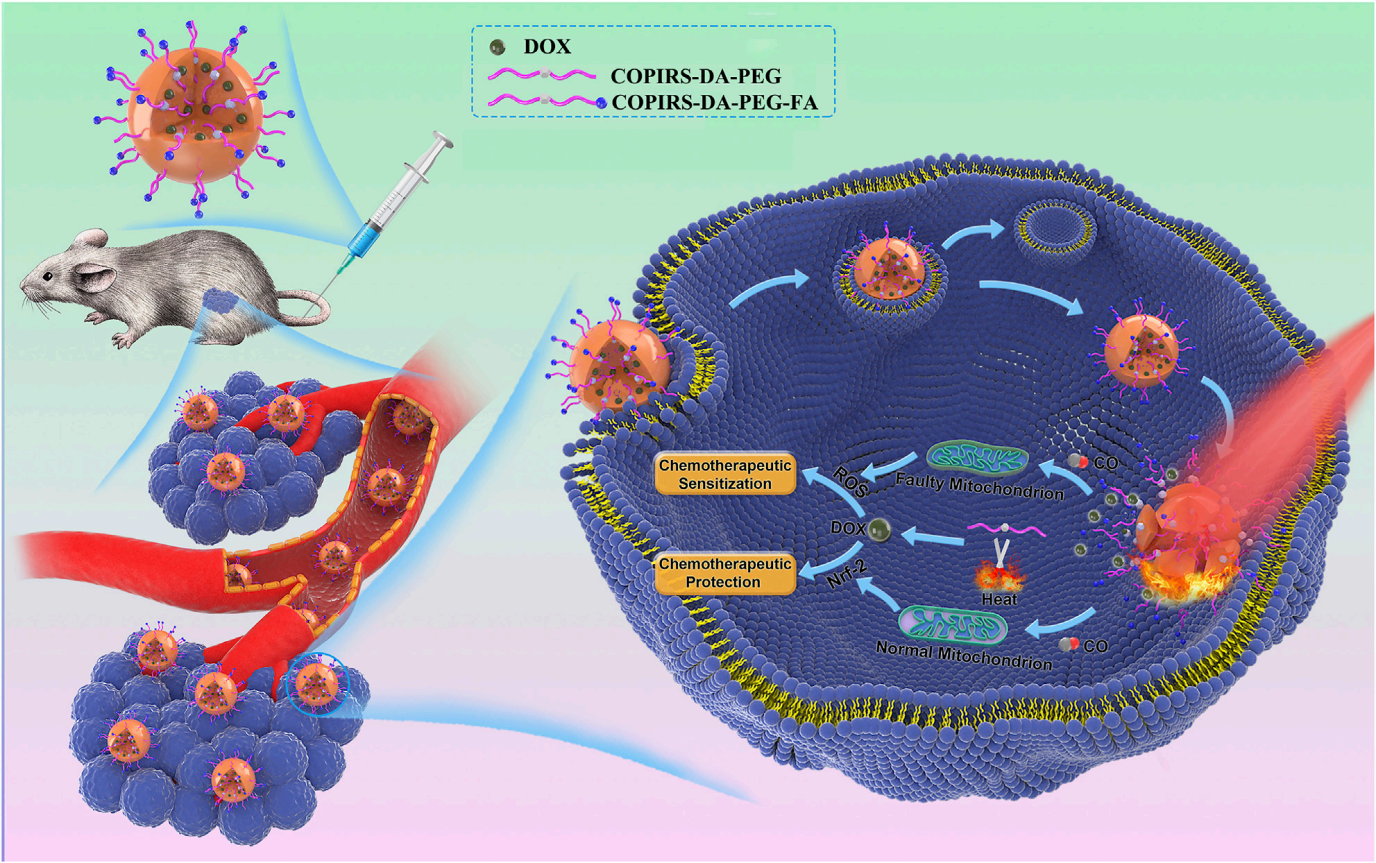
After accumulation in the tumor and endocytosis by tumor cells, the nanomedicine could synergistically control the release of CO and drugs under NIR irradiation. CO can distinguish between tumor and normal cells through differences between their mitochondria and regulates their resistance, simultaneously achieving an enhancement of the efficacy of chemotherapy drugs on tumor cells and chemotherapeutic protection on normal cells.

Herein, we report a tumor/normal cells distinguishing nanomedicine (CO&Dox@NPs) that is able to synergistically release CO and chemotherapeutic drugs under near-infrared (NIR) light irradiation, so as to realize both CS and CP in a unified system. Owing to the merits that fluorescent dye (IR808) can produce active electrons (Wu et al., 2015) and heat (Jung et al., 2018; Wei et al., 2020) under NIR light, the CO photo-induced release system (COPIRS) was constructed by the coupling of IR808 with MnBr(CO)_5_ (He et al., 2015; Wu et al., 2018), and the photothermal-induced drug release system was achieved by introducing a thermal-responsive groups (DA group) between hydrophilic polymer (polyethylene glycol; PEG) and COPIRS through the Diels–Alder reaction to form an amphiphilic copolymer (COPIRS-DA-PEG) (Bakhtiari et al., 2009; Zandberg et al., 2012). CO&Dox@NPs possess the ability of burst-releasing under NIR light, which could be matched with the rapid release of CO induced by active electrons. Thus, the CO&Dox@NPs were endowed with the function of synergistically controlled release of CO and drugs (Figure 1). CO&Dox@NPs could greatly improve the anti-tumor effect both *in vitro* and *in vivo*, and it could also protect normal cells with the results of increasing the drug resistance of human umbilical vein endothelial cells (HUVEC) by nearly 1,000 fold. We believe that such tumor/normal cells distinguishing strategy would improve the clinical practice tumor chemotherapy.

## Experimental Section

### Materials

Maleimide-functionalized PEG (mPEG-Mal) (Mw ≈ 2 kDa) and t-Boc-NH-PEG-Mal (Mw ≈ 2 kDa) were purchased from JenKem Technology Co. Ltd (Beijing, China). Na_3_C_6_H_5_O_7_·2H_2_O, CuCl_2_, Na_2_S, cysteamine, 4,4′-diamino-2,2′-bipyridine (DABPY), tetra-n-butylammonium bromide, furfurylamine were purchased from Energy Chemical (Shanghai, China). MnBr(CO)_5_, 1-(3-Dimethylaminopropyl)-3-ethylcarbodiimide hydro (EDC), N-hydroxysulfosuccinimide sodium salt (sulfo-NHS), myoglobin, and folic acid (FA) were purchased from Sigma-Aldrich (St. Louis, MO, United States). All reagents used were of analytical grade. The water used in all experiments was deionized (DI) water with a resistivity of 18.2 MΩ cm. The molecular weight cutoff of all dialysis bags used in the dialysis process was of 5 kDa. High-glucose DMEM containing 1% penicillin/streptomycin, phosphate-buffered saline (PBS), and trypsin was obtained from KeyGen BioTech Co. Ltd. (Jiangsu, China). Fetal bovine serum was purchased from Absin Bioscience Inc. (Shanghai, China).

### Synthesis of CO Photo-Induced Release System (COPIRS)

In total, 0.38 g of IR808 (0.5 mmol), 0.19 g of EDC, and 0.22 g of sulfo-NHS were dispersed in 20 ml of N,N-dimethylformamide (DMF). Then 0.45 g of DABPY was added and stirred overnight at room temperature. BPY-IR-BPY was obtained after dialysis and lyophilization.

Next, 0.46 g of IR808 (0.6 mmol), 0.12 g of EDC, and 0.13 g of sulfo-NHS were dispersed in 30 ml DMF. Then 0.27 g of BPY-IR-BPY (0.25 mmol) was added and stirred overnight at room temperature. Then 0.14 g of MnBr(CO)_5_ was added and the solution was stirred at room temperature for 24 h under an N_2_ atmosphere. COPIRS was obtained after dialysis and lyophilization.

### Characterization of COPIRS

Composition and morphology characterization: Fourier transform infrared (FT-IR) spectroscopy analysis was performed by mixing sample (IR808, MnBr(CO)_5_, DABPY, and COPIRS) powder with KBr (5 mg: 1 g W/W). The elemental composition was assessed by XPS (ThermoFisher K-Alfa) with a focused monochromatic Al X-ray (1,486.6 eV) source. UV-vis spectra of IR808 and COPIRS solution (the solvent is DMF) were measured by enzyme-labeled instrument (Biotek Eon™).

The CO controlled release ability of COPIRS was assessed by measuring the conversion of deoxy myoglobin (deoxy-Mb) to carbonmonoxy myoglobin (MbCO) as previously reported (Motterlini et al., 2002). Myoglobin (Mb) was dissolved in PBS (pH 6.8) at a concentration of 0.66 μM and the Mb was converted to deoxy-Mb by adding sodium dithionite (0.1%). COPIRS (5 mg/ml) was then added and exposed to laser irradiation (808 nm, 0.78 W/cm^2^). The change of absorption spectra was measured by enzyme-labeled instrument (Biotek Eon™).

Photocurrent measurements were performed using a three-electrode configuration, with pure phase IR_3_BPY_2_ and COPIRS films as the working electrode, saturated Ag/AgCl as the reference electrode, and a platinum mesh as the counter electrode. The working electrode films were prepared by spin-coating the ethanol solution of the sample onto indium–tin oxide (ITO) to form a film. Linear sweep voltammetry (LSV) measurements were performed in the absence of light and under NIR light irradiation (λ ∼ 808 nm) using a 0.5 M Na_2_SO_3_ solution at an open circuit voltage over a potential range from −0.3 to 0.1 V.

### Synthesis of COPIRS-DA-PEG

mPEG-DA was prepared by the Dies–Alder reaction, wherein 2.0 g of mPEG-Mal were dissolved in DMF and 1.1 ml of furfurylamine were added, stirred at room temperature in an N_2_ atmosphere for 48 h, and purified by dialysis and lyophilization.

COPIRS-DA-PEG was then prepared by an amide reaction, wherein 0.3 g of COPIRS, 0.19 g of EDC and 0.22 g of sulfo-NHS were dissolved in 10 ml of DMF, and then 0.2 g of mPEG-DA was added. The mixture was stirred at room temperature overnight. COPIRS-DA-PEG was purified by dialysis and lyophilization.

### Characterization of COPIRS-DA-PEG

FT-IR of mPEG-Mal, mPEG-DA, COPIRS-DA-PEG were performed as mentioned above. ^1^H-NMR spectra of mPEG-DA and COPIRS-DA-PEG were obtained from a Bruker ^1^H-NMR 400 DRX Spectrometer. COPIRS-DA-PEG was dissolved in water and incubated at 65°C for 10 min and then dialyzed against DI water and lyophilized. The molecular weight of the powder at pre- and post-incubation was measured by an Agilent Gel Permeation Chromatography (GPC) system with tetrahydrofuran as the mobile phase and calculated according to the PEG standards. ^1^H-NMR spectra of these two products were obtained from a Bruker ^1^H-NMR 400 DRX Spectrometer.

### Synthesis of COPIRS-DA-PEG-FA

In order to endow the nanomedicine with the function of tumor targeting, the nanomedicines are surface-functionalized with FA to promotes the nanomedicine’s entry into cells through folate receptor-mediated endocytosis, which is overexpressed outside tumor cells. t-Boc-NH-PEG-DA-COPIRS was prepared as mentioned above (2.4). t-Boc-NH-PEG-DA-COPIRS was then de-protected by trifluoroacetic acid (TFA) into COPIRS-DA-PEG-NH_2_ as previously reported (Yu et al., 2015), by dissolving 0.2 g of t-Boc-NH-PEG-DA-COPIRS in a mixed solution of 10 ml of dichloromethane and 2 ml of TFA at 0°C for 45 min. Dichloromethane and TFA were then removed by evaporation and the NaHCO_3_ solution (1 M) was added to stop the reaction. Finally, 0.1 g of COPIRS-DA-PEG-NH_2_, 11.0 mg of FA, 19.2 mg of EDC, and 11.5 mg of sulfo-NHS were dissolved in 10 ml of dichloromethane and stirred overnight at room temperature. COPIRS-DA-PEG-FA was obtained after dialysis and lyophilization.

### Preparation of CO&Dox@NPs

CO&Dox@NPs were prepared by a solvent evaporation method as previously described (Hu et al., 2007; Lee et al., 2010; Yuan et al., 2017; Liu et al., 2019). Briefly, 45 mg of COPIRS-DA-PEG, 5 mg of COPIRS-DA-PEG-FA, and 5 mg of Dox were dissolved in 5 ml of methylene chloride and then added to 50 ml of DI water. The mixture was emulsified by ultrasound for 10 min and methylene chloride was then removed through a rotary evaporator. Following centrifugation and lyophilization, CO&Dox@NPs were obtained.

IR_3_BPY_2_-DA-PEG@NPs, CO@NPs, and Dox@NPs were prepared by the above method, with the differences being that only 5 ml of dichloromethane were added:

IR_3_BPY_2_-DA-PEG@NPs: 45 mg of IR_3_BPY_2_-DA-PEG, 5 mg of IR_3_BPY_2_-DA-PEG -FA
CO@NPs: 45 mg of COPIRS-DA-PEG, 5 mg of COPIRS-DA-PEG-FA
Dox@NPs: 45 mg of IR_3_BPY_2_-DA-PEG, 5 mg of IR_3_BPY_2_-DA-PEG -FA, 5 mg of Dox

### Characterization of CO&Dox@NPs

Photothermal conversion: Absorption spectra were recorded with a UV-vis spectrophotometer (Beijing Persee DU1900). Temperature variation profiles of the COPIRS were obtained by measuring the change in temperature of a water and CO&Dox@NPs solution (1 mg/ml) exposed to laser irradiation (808 nm, 0.78 W/cm^2^).

Photothermal response of CO&Dox@NPs: After the CO&Dox@NP solution was irradiated by 808 nm laser for 10 min, Transmission electron microscopy (TEM, JEOL JEM-2010 microscope) were performed to observe the changes in particle size and morphology. The CO&Dox@NPs solution without laser irradiation was also observed.

CO-loading content: CO is demarcated by a carbonyl group, which is three times that of Mn atom according to molecular structure formula. Ten milligrams of CO&Dox@NPs (weight of CO&Dox@NPs (W_CO&Dox@NPs_) = 10 mg) were dissolved in aqua regia, heated at 90°C until complete dissolution, and the solution was fixed to a volume of 2 ml. The concentration of Mn (C_Mn_) was tested by ICP-MS. The CO-loading content was calculated according to the following Eq. 1:

$$\mathrm{CO loading content}=\frac{C_{\mathrm{Mn}}\times 2\mathrm{mL}}{W_{\mathrm{CO}\&\mathrm{Dox}@\mathrm{NPs}}}\times \frac{3\times 28g/\mathrm{mol}}{55g/\mathrm{mol}}\times 100\%$$ (1)

Dox-loading content: 10 mg of CO&Dox@NPs (W_CO&Dox@NPs_ = 10 mg) were dissolved in 10 ml of acetonitrile and PBS (pH 7.4) (V/V = 2:3) mixed solvent and the supernatant was centrifuged. The Dox concentration (C_Dox_) was measured by absorption photometry. The Dox-loading content was calculated by Eq. 2:

Dox loading content=CDox×10mL WCO&Dox@NPs×100% (2)

The CO controlled release ability of CO&Dox@NPs was measured through the deoxy-Mb carbonylation assay as mentioned above.

The Dox controlled release ability of CO&Dox@NPs was measured using the following method: 2 mg of CO&Dox@NPs were dispersed in 5 ml of PBS (pH 7.4) and encapsulated in a dialysis bag and then immersed in 45 ml of PBS (pH 7.4). Two samples were prepared; one was treated in the dark and the other was irradiated with an 808 nm laser for 10 min and then placed in a 37°C thermostatic oscillator. At pre-determined time intervals, 100 μl of the release medium were removed. The ultraviolet absorption of the released medium was measured at 480 nm.

### Cell Culture

HUVEC and mouse breast cancer cell line 4T1 (4T1) were purchased from the American Type Culture Collection. DMEM was supplemented with 10% fetal bovine serum and 1% penicillin and streptomycin. Cells were cultured and maintained in the above-mentioned growth medium and incubated overnight at 37°C in 5% CO_2_ at 95% humidity (MCO-18AC, Panasonic).

### *In Vitro* Cytotoxicity Assay

The cytotoxicity of nanocarrier (CO@NPs) was measured by the MTT (3-(4,5-dimethylthiazol-2-yl)-2,5-diphenyltetrazolium bromide) assay. HUVEC and 4T1 cells were cultured in 96-well plates until 80–85% confluence. Subsequently, 100 μl of the fresh medium containing different concentrations of the CO@NPs were added to replace the medium. After 24 h, the medium was removed and the cells were washed twice with saline; 200 μl of the MTT solution (2.5 mg/ml, soluble in PBS) were added and the cells were incubated for another 4 h. The medium was then siphoned and the cells were re-dispersed in 200 μl of DMSO. Their absorbance was measured at 490 nm.

### Intracellular Controlled Release of CO

The intracellular controlled release of CO was detected by the fluorescent molecule COP-1. COP-1 was synthesized as reported (Michel et al., 2012). A sample of 4T1 cells were co-cultured with the medium containing IR_3_BPY_2_-DA-PEG@NPs (group 1), CO&Dox@NPs (group 2), CO&Dox@NPs (group 3), or COPIRS (group 4) for 4 h in the dark and then replaced with the medium containing COP-1 (5 mg/ml) for another 1 h. Groups 1, 2, and 4 were irradiated by 808 nm laser for 10 min and the fluorescence of the cells was subsequently observed using a laser scanning confocal microscope.

### *In Vitro* Tumor Suppressive Effect

Then, 4T1 cells were cultured in 96-well plates. When cells proliferated to ∼80–85% of confluence in every well, 100 μl of the medium containing different samples was added to each well: (1) IR_3_BPY_2_-DA-PEG@NPs, (2) Dox@NPs, (3) CO@NPs, (4) CO&Dox@NPs, and (5) CO&Dox@NPs. Groups 3 and 5 were irradiated by 808 nm laser for 10 min at 6 h post the incubation. After another 18 h of culture in the dark, the MTT assay was used to measure cell viability in each group.

HUVEC cells were also incubated in 96-well plates and 100 μl of the medium containing IR_3_BPY_2_-DA-PEG@NPs, CO&Dox@NPs, Dox@NPs was added. The last two groups were irradiated by 808 nm laser for 10 min at 6 h post the incubation. After another 18 h of culture in the dark, the MTT assay was used to measure cell viability in each group.

### Tumor Model

A mouse breast cancer model was established by the following method: 5 × 10^5^ 4T1 cells (100 μl) were subcutaneously injected into the second fat pad of the right breast of BALB/C mice (female, 4–6 weeks old, ∼20 g) and the mice were fed until the tumor volume grew to ∼100 mm^3^ for further experiments.

All animal protocols were reviewed and approved by the committee on animals in Nanjing University and carried out according to the guidelines provided by the National Institute of Animal Care.

### *In Vivo* Fluorescence Imaging

As IR808 is a fluorescent dye, there is no need for additional fluorescent dye labeling. CO&Dox@NPs were dispersed in PBS (pH = 7.3) and 100 μl of CO&Dox@NPs suspension (10 mg/ml) was administrated to 4T1 tumor-bearing nude mouse via tail vein injection. *In vivo* fluorescence images were obtained before injection and 1, 3, 5, 8, 10, and 24 h post injection (Ex: 808, Em: 830).

### *In Vivo* Tumor-Suppressive Effect

When the tumor size reached ∼100 mm^3^, mice were divided into six groups (*n* = 5 per group). Each mouse was intravenously injected with 100 μl of the various samples: normal saline (Cont; group 1), IR_3_BPY_2_-DA-PEG@NPs (200 mg/kg; group 2), Dox@NPs (200 mg/kg; group 3), CO@NPs (200 mg/kg; group 4), CO&Dox@NPs (220 mg/kg; group 5), CO&Dox@NPs (220 mg/kg; group 6), respectively, and groups 4 and 6 were treated with 808 nm laser for 10 min at 6 h post the injection. The treatment was given twice a week for 2 weeks. The length (L, longest diameter) and width (W, shortest diameter) of the tumor were measured with Vernier calipers every 2 days from the beginning of treatment. The tumor volume (V) was calculated according to Eq. 3:

$$V=\left(L\times W^{2}\right)/2$$ (3)

The survival of mice was counted every 2 days.

Two other mice were prepared and treated as the group (1) and group (6), and they were sacrificed at 10th day, that is, after the treatment. Tumors were harvested, isolated, immobilized, embedded into paraffin, cut into sections, stained by H&E, and observed with a microscope.

### Pathological Analysis and Hematological Assay

Tumor-bearing mice were intravenously injected with CO&Dox@NPs and irradiated with 808 nm laser for 10 min and then sacrificed 1, 7, and 14 days later. Mice without any treatment served as the control group (Cont). The sections of main organs (heart, liver, spleen, lung, kidney) stained by H&E were harvested and observed with a microscope. Blood was collected in sodium EDTA anticoagulant tubes and analyzed.

## Results and Discussion

### Synthesis and Characterization of COPIRS

**FIGURE 2:**
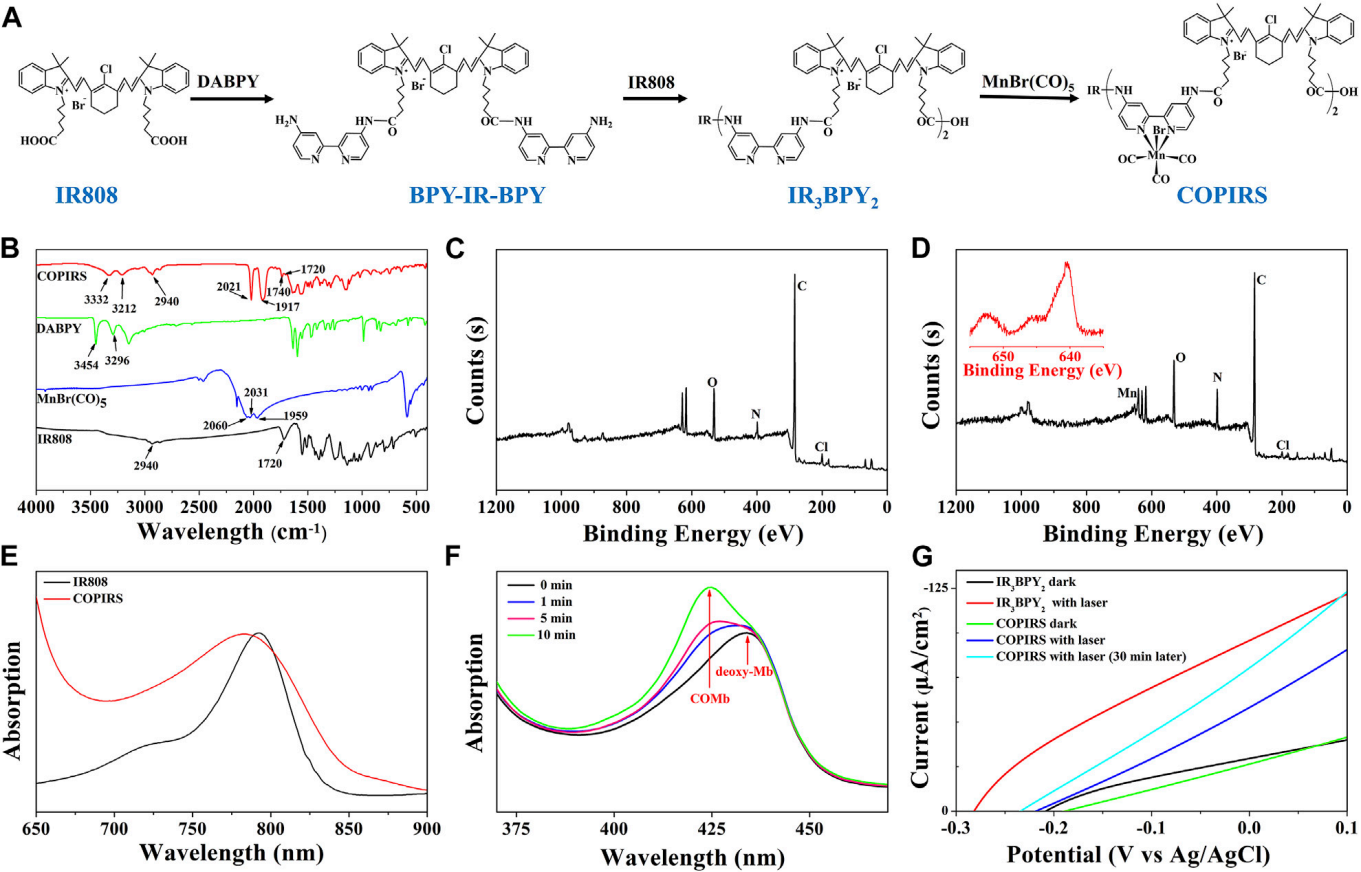
Characterization of CO photo-induced release system (COPIRS); **(A)** The synthetic route of COPIRS. **(B)** FT-IR spectra of IR808, MnBr(CO)_5_, DABPY, and COPIRS; **(C)** XPS result of IR808; **(D)** XPS result of COPIRS (inset: amplification of Mn (red) peak); **(E)** UV-vis spectra of IR808 and COPIRS; **(F)** UV-vis spectra of deoxy-Mb solution co-incubated with COPIRS before NIR irradiation and at 1, 5, and 10 min after NIR irradiation (MbCO: carbonmonoxy myoglobin); **(G)** Photocurrent measurements of IR_3_BPY_2_ in dark and with laser (808 nm), COPIRS in dark, with laser (808 nm), and with laser (808 nm) after irradiation of 30 min through linear sweep voltammetry (LSV) measurements.

As shown in Figure 2, COPIRS was synthesized by attaching bipyridine and carbonyl manganese to fluorescent dye (IR808) to construct the electron excitation–electron transfer group–electron-induced CO release structure (He et al., 2015; Wu et al., 2018). The successful synthesis of COPIRS from IR808 was demonstrated by FT-IR and XPS spectroscopy. In the FT-IR spectra (Figure 2B), the peaks at 3454 and 3296 cm^−1^ assigned to the N-H stretching vibration of amino in DABPY disappeared, and the peaks at 3332 and 3212 cm^−1^, attributed to the N-H of the amide bond, appeared. Also the peak at 1740 cm^−1^ assigned to the C=O stretching vibration of amide bond in COPIRS appeared. These changes indicate the reaction between the carboxyl groups from IR808 and amino groups from DABPY. The peaks at 2940 and 1720 cm^−1^ assigned to the O-H and C=O stretching vibration of carboxyl group still exists in COPIRS, corresponding to the carboxyl group at either end COPIRS. A comparison of the spectra of MnBr(CO)_5_ and COPIRS showed the absorption peak of C≡O shifted toward a lower frequency from 2060, 2031, and 1959 cm^−1^ to 2021 and 1917 cm^−1^, indicative of the loss of part C≡O and a weakened bonding of C≡O, suggesting the partial replacement of C≡O coordinated on Mn by the bipyridine group as a worse π-acceptor ligand. This evidence indicates that -MnBr(CO)_3_ was attached to IR_3_BPY_2_ and thus the formation of COPIRS. In the XPS curve, the presence of Mn peak in COPIRS further indicated the successful connection of -MnBr(CO)_3_ to IR808 (Figures 2C,D).

The UV-vis absorption spectrum of COPIRS was the measured to illustrate that COPIRS can be excited by NIR light. The absorption peak of COPIRS was at 785 nm and still has strong absorption value at 808 nm (Figure 2E), meaning that it can be excited by light at 808 nm. The ability of COPIRS to release CO under the NIR light irradiation was characterized by evaluating the conversion of deoxy-Mb to MbCO (Motterlini et al., 2002). After 1 min of laser irradiation, the absorption peak intensity of deoxy-Mb (434 nm) decreased and transformed into an absorption peak that appeared at 425 nm, indicating the production of MbCO (Figure 2F). After 10 min of laser irradiation, the absorption peaks at 554 nm disappeared and the absorption peaks appeared at 540 and 577 nm, which could further prove the release of CO from COPIRS under NIR light irradiation (Supplementary Figure S1).

In previous reports, the researchers hypothesized that active electrons could induce the release of CO from manganese carbonyl group. On this basis, we speculate that the release of CO from COPIRS was induced by photoelectron, which was produced by IR808 excited under NIR light. To test this hypothesis, photocurrent of IR_3_BPY_2_ and COPIRS were measured by LSV measurements. As shown in Figure 2G, the photocurrent of COPIRS changed from in dark to with laser (808 nm) was much lower than that of IR_3_BPY_2_, which was because of the consumption of photoelectron of COPIRS to release CO. After 30 min of laser irradiation, the photocurrent was greatly enhanced, because of loss of C≡O from -MnBr(CO)_3_ and decrease of photoelectron consumption. This evidence suggests that the release of CO from COPIRS was excited by photoelectron.

### Synthesis and Characterization of COPIRS-DA-PEG

**FIGURE 3:**
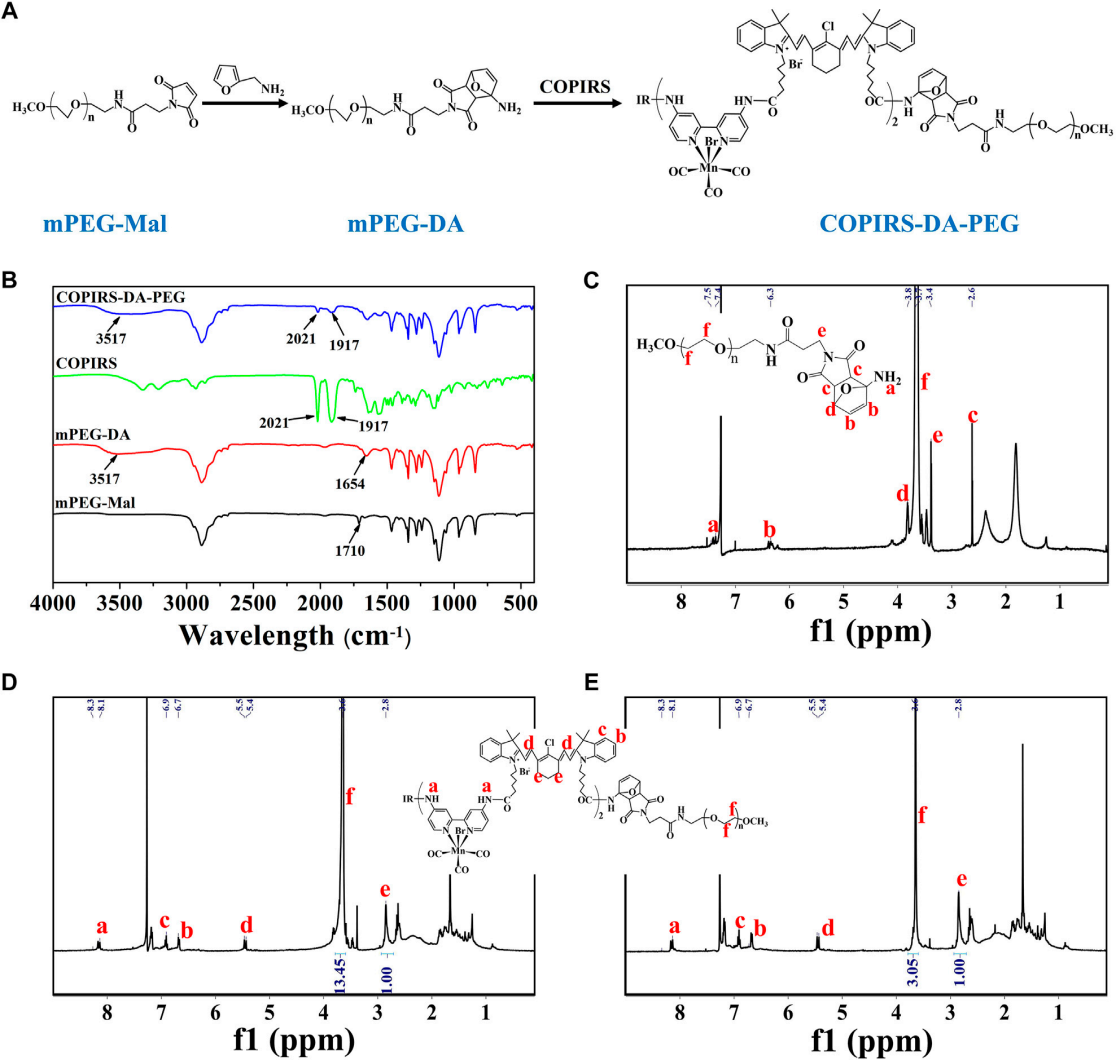
Characterization of COPIRS-DA-PEG. **(A)** The synthetic route of COPIRS-DA-PEG. n≈41; **(B)** FT-IR spectra of mPEG-Mal, mPEG-DA, COPIRS, and COPIRS-DA-PEG; **(C)** ^1^H-NMR spectrum of mPEG-DA; **(D)** ^1^H-NMR spectrum of COPIRS-DA-PEG; **(E)** ^1^H-NMR spectrum of COPIRS-DA-PEG after incubation at 65°C and dialysis.

The molecular structure and synthetic route of COPIRS-DA-PEG is shown in Figure 3A. The thermally responsive DA group was formed by the Dies–Alder reaction between furan and maleimide. In order to verify the formation and thermal response ability of COPIRS-DA-PEG, its structure was characterized by FT-IR (Figure 3B) and ^1^H-NMR (Figures 3C–E) and its molecular weight was assessed by GPC (Table 1).

**TABLE 1:** Molecular weight (M_w_) of COPIRS-DA-PEG after different treatments.

Sample	EO/IR (feed)^a^	EO/IR (product)^b^	M_w_ ^b^ (g/mol)	M_w_ ^d^ (g/mol)	M_w_ ^a^ (g/mol)
COPIRS -DA-PEG	13.67	13.45	5000	4980	5030
COPIRS -DA-PEG after heating	0	3.05	3630	3420	3030 (M_w, COPIRS_)

a Determined by feed.

b Determined by.

c H-NMR.

d Determined by GPC.

The characteristic peaks around 3,517 cm^−1^ attributed to the N-H stretching vibration absorption of amino appeared in mPEG-DA. Also the peak at 1710 cm^−1^ assigned to the maleimide group in mPEG-Mal disappeared and the peak at 1654 cm^−1^ assigned to the DA group appeared, which indicates the successful synthesis of mPEG-DA (Figure 3B). The formation of the DA group was then further proved by ^1^H-NMR in Figure 3C, H atoms on the DA group could be found in their corresponding positions. In the meantime, the characteristic absorption peaks of mPEG-DA at 3517 cm^−1^ and 1654 cm^−1^ and COPIRS at 2021 cm^−1^ and 1917 cm^−1^ appeared in COPIRS-DA-PEG in FT-IR (Figure 3B), and H atoms on PEG and COPIRS appeared in their corresponding positions in COPIRS-DA-PEG in ^1^H-NMR (Figure 3D), which indicates the successful synthesis COPIRS-DA-PEG. The ratio of IR to PEG monomers (ethylene oxide; EO) was equal to 1:13.45 (calculated by the area ratio of *e* to *f*), conforming to the polymer composition (3 IR on each COPIRS and 41 EO on each PEG). After 10 min of incubation at 65°C, the intensity of the peak PEG weakened (the area ratio of *e* to *f* went from 1:13.45 to 1:3.05), demonstrating the broken of DA group and the loss of PEG after dialysis (Figure 3E). These results indicated that COPIRS-DA-PEG was successfully synthesized and the breakage of the DA group at high temperatures (above 65°C).

The molecular weight of COPIRS-DA-PEG was measured to confirm the conjugation of COPIRS and PEG and the formation of the DA group. The molecular weight of as-synthesized COPIRS-DA-PEG and COPIRS-DA-PEG experienced heating at 65°C were measured by GPC and calculated by ^1^H-NMR (Table 1). The EO:IR molar ratio in COPIRS-DA-PEG was determined from the area ratio of the peak f to peak e in the ^1^H-NMR spectra, and the molecular weight was calculated according to Eq. 4.

$${\bar{M}}_{W}={\bar{M}}_{W,\mathrm{COPIRS}}+44{\bar{\mathrm{DP}}}_{\mathrm{PEG}}+196$$ (4)

where $\;{\bar{DP}}_{PEG}=3\times \left(EO/IR\right)$ , 3 is the amount of IR on COPIRS, and 196 is the molecular weight of the group remaining in mPEG-Mal except EO. The molecular weight of COPIRS-DA-PEG as measured by GPC and calculated by ^1^H-NMR was 4.98 and 5.00 kDa, respectively, which is close to the theoretical value calculated from the feed ratio indicating the successful formation of COPIRS-DA-PEG. After 10 min of incubation at 65°C and dialysis, the molecular weight was 3.42 and 3.63 kDa, respectively, as determined by GPC and ^1^H-NMR, thus close to the molecular weight of COPIRS, indicating the breakdown of COPIRS-DA-PEG under heating and the loss of water-soluble PEG after dialysis.

### Preparation and Characterization of CO&Dox@NPs

**FIGURE 4:**
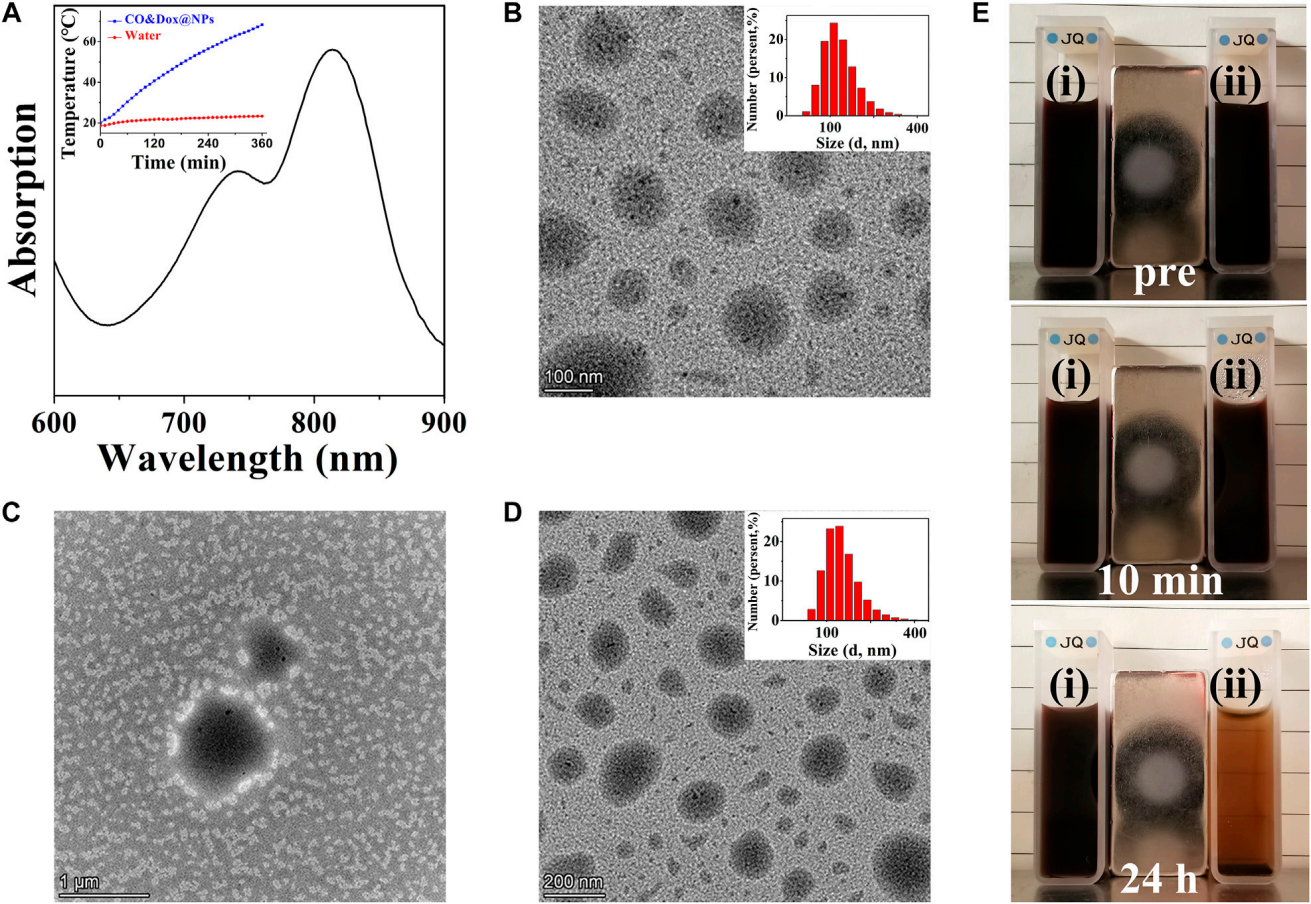
Characterization of nanomedicine (CO&Dox@NPs). **(A)** UV-vis spectra of CO&Dox@NPs solution (insert: temperature variation profiles of the CO&Dox@NPs); **(B)** TEM image of freshly prepared CO&Dox@NPs (inset: hydrodynamic diameter distribution); **(C)** TEM image of freshly prepared CO&Dox@NPs after 10 min of irradiation; **(D)** TEM image of CO&Dox@NPs after 24 h of standing without irradiation (inset: hydrodynamic diameter distribution); **(E)** Optical camera image of CO&Dox@NPs with (i) and without (ii) NIR irradiation, pre-irradiation (pre), after 10 min of irradiation (10 min), and 24 h post irradiation (24 h).

CO&Dox@NPs were formed by the self-assembly of the amphiphilic block copolymer COPIRS-DA-PEG in selective solvent. Given that COPIRS-DA-PEG exhibited thermally responsive behavior and that the encapsulated COPIRS exhibited photothermal conversion behavior, it was posited that CO&Dox@NPs would possess a photo-responsive ability and would be disintegrated under NIR irradiation. To verify this hypothesis, the UV-Vis absorption and the photothermal conversion ability of CO&Dox@NPs was measured. As shown in Figure 4A, CO&Dox@NP solution exhibits strong absorptive capacity at 808 nm, and the temperature variation profiles showed that the temperature of CO&Dox@NP solution (1.0 mg/ml) increased by 48.3°C in 6 min, reaching 68.3°C. This means that CO&Dox@NPs has a photothermal conversion capability, and the photothermal was enough to induce the decomposition of COPIRS-DA-PEG.

The morphology of CO&Dox@NPs under and without NIR irradiation was observed by TEM (Figures 4B–D). CO&Dox@NPs and showed a uniform size and good dispersion in aqueous solution (Figure 4B). Their mean hydrodynamic diameter, as measured by DLS, was 166 nm with a polydispersity index of 0.084 (insert). After 10 min of NIR irradiation, the boundary of CO&Dox@NPs was no longer visible and only polymer fragment assembly could be observed, indicating that the nanoparticles were completely disintegrated (Figure 4C). However, the CO&Dox@NPs standing 24 h without irradiation showed minimal change and retained a good water dispersibility (Figure 4D), with a mean hydrodynamic diameter of 172 nm as measured by DLS and a polydispersity index of 0.102 (Figure 4D, insert). In order to demonstrate the photothermal degradation effect of nanocarriers, the CO@NPs solution treated under and without NIR irradiation were observed with the optical camera (Figure 4B). After 10 min of laser irradiation and 24 h of standing, precipitate appeared and the solution became clear. The solution of CO@NPs without irradiation did not change after 24 h of standing.

These results show that the nanomedicine (CO&Dox@NPs) was successfully prepared and that it possesses a photo-responsive ability. Under NIR irradiation, COPIRS generates heat, leading to the decomposition of COPIRS-DA-PEG, the loss of its stable structure, and subsequent disintegration. Internal thermal expansion would then cause the collapse of nanomedicine quickly, within the 10 min of NIR irradiation.

### Encapsulation and *In Vitro* Release of CO and Dox

**FIGURE 5:**
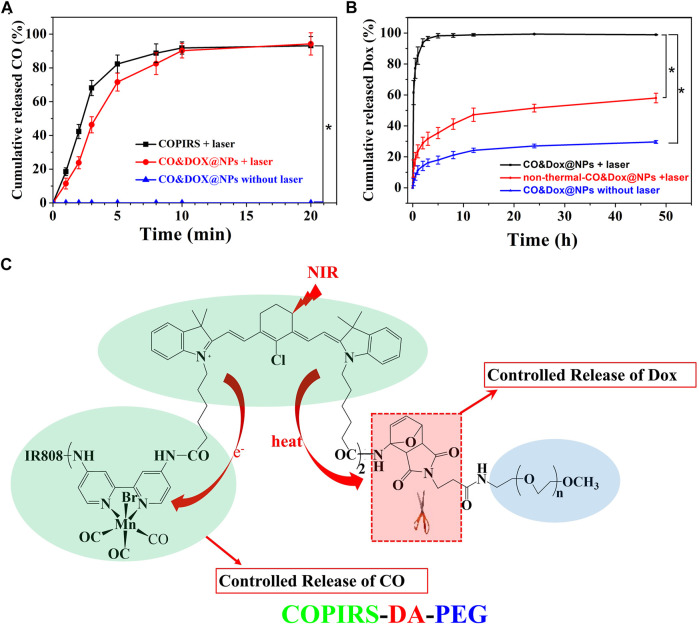
*In vitro* release probe. **(A)** *In vitro* release of CO from COPIRS and CO&Dox@NPs with and without NIR laser irradiation. NIR laser (808 nm) irradiation continued during the test from 0 min to 20 min. **(B)** *In vitro* release of Dox from CO&Dox@NPs with and without NIR laser irradiation and none-thermally responsive none-thermal-CO&Dox@NPs with irradiation; 10 min of NIR laser (808 nm) irradiation were performed before the test. *p < 0.001. **(C)** The mechanism of the spatial and temporal consistency of the controlled release of CO and drug under near-infrared light (NIR, 808 nm).

The CO-loading content (3.12%) was calculated by measuring the concentration of Mn by ICP-MS because CO is demarcated by the carbonyl group, which is three times of Mn atom according to molecular formula (Table 2). As COPIRS can release CO when irradiated (Figure 2G), we speculated that CO&Dox@NPs also had a CO photo-releasing ability. Thus, the cumulative *in vitro* release of CO from COPIRS and CO&Dox@NPs under NIR laser irradiation and that of CO&Dox@NPs without NIR laser irradiation was measured (Figure 5A). During the first 5 min of irradiation, ∼71.6 ± 5.3% of the total CO content was released. After 10 min of irradiation, ∼90.2 ± 4.3% of the total CO content was released. These values are relatively lower than that of COPIRS, presumably due to the blocking effect of the nanocarrier on CO diffusion. In contrast, almost no CO was detected in CO&Dox@NPs without irradiation within 20 min, proving that CO&Dox@NPs can undergo the NIR-controlled release of CO.

**TABLE 2:** CO- and Dox-loading content.

	Concentration measured (μg/ml)	Loading content calculated according to formula (wt%)	Loading content calculated according to feed (wt%)
CO	C_Mn_ = 102.3	3.12	3.34
Dox	C_Dox_ = 88.2	8.82	9.09

The Dox-loading content (8.82%) was determined by an UV-vis spectroscopy because the concentration of Dox was proportional to the absorbance value at 480 nm (Table 2). The cumulative *in vitro* release of Dox from CO&Dox@NPs with or without NIR laser irradiation was measured (Figure 5B). The release rate of Dox from CO&Dox@NPs without irradiation was relatively slow. Only 5.2% of Dox was released into the medium within the initial 10 min and 24.5% of Dox was released after 24 h. The initial burst release of Dox could be ascribed to the Dox adhered onto the surface of CO&Dox@NPs. However, for the CO&Dox@NPs, after 10 min of laser irradiation, ∼61.5 ± 7.8% of Dox was released at 10 min; after 24 h, most of the Dox (99.3 ± 0.2%) was released. These data demonstrated that laser irradiation could control the release of Dox because of the presence of the thermally responsive DA groups in CO&Dox@NPs. To further verify the thermally responsive effect of DA groups on the controlled release of Dox, COPIRS-PEG (without DA groups, which was prepared by the reaction of mPEG-NH_2_ with COPIRS), instead of COPIRS-DA-PEG, was used to fabricate the nanomedicine (named as none-thermal-CO&Dox@NPs). The release of Dox from none-thermal-CO&Dox@NPs was monitored under NIR irradiation (Figure 5B). About 12.4 ± 5.6% and 51.5 ± 2.5% of Dox were released at 10 min and 24 h, respectively. Thus, the release rate of Dox from none-thermal-CO&Dox@NPs was faster than that from CO&Dox@NPs without laser irradiation and slower than that of CO&Dox@NPs with laser irradiation. This phenomenon may be related to the presence of DA groups in the CO&Dox@NPs. Under laser irradiation, the internal COPIRS within CO&Dox@NPs could transform light to heat, breaking the DA groups and resulting in the disintegration of CO&Dox@NPs. Therefore, the rate of Dox release would be accelerated. Conversely, for the none-thermal-CO&Dox@NPs, following laser irradiation, COPIRS also transformed light to heat and increased the temperature of none-thermal-CO&Dox@NPs. Due to the absence of DA groups, none-thermal-CO&Dox@NPs maintained their integrated morphology but with a loosening of the structure because of the high temperature. Consequently, none-thermal-CO&Dox@NPs would release Dox more slowly than CO&Dox@NPs. However, without laser irradiation, CO&Dox@NPs did not exhibit a photo-responsive ability, leading to the slowest release rate.

These results further affirmed the photo-responsive ability of CO&Dox@NPs and that the disintegration of CO&Dox@NPs resulted in the quick release of Dox from CO&Dox@NPs a short time after NIR irradiation.

Based on above results, we concluded that both CO and Dox could be released from CO&Dox@NPs within a short time after NIR irradiation, ensuring their synergistic release (Figure 5C). When these CO&Dox@NPs were internalized by tumor cells, CO and Dox would be released simultaneously inside the tumor, maximizing the CS effect on Dox against tumor cells and leading to an enhanced chemotherapy outcome. When these CO&Dox@NPs were in the normal cells, the presence of CO would protect these normal cells from being damaged by Dox, consequently reducing the side effect of Dox. Therefore, these CO&Dox@NPs not only showed a good anti-tumor effect but also had less toxicity to health tissue with laser irradiation.

### *In Vitro* Analysis

**FIGURE 6:**
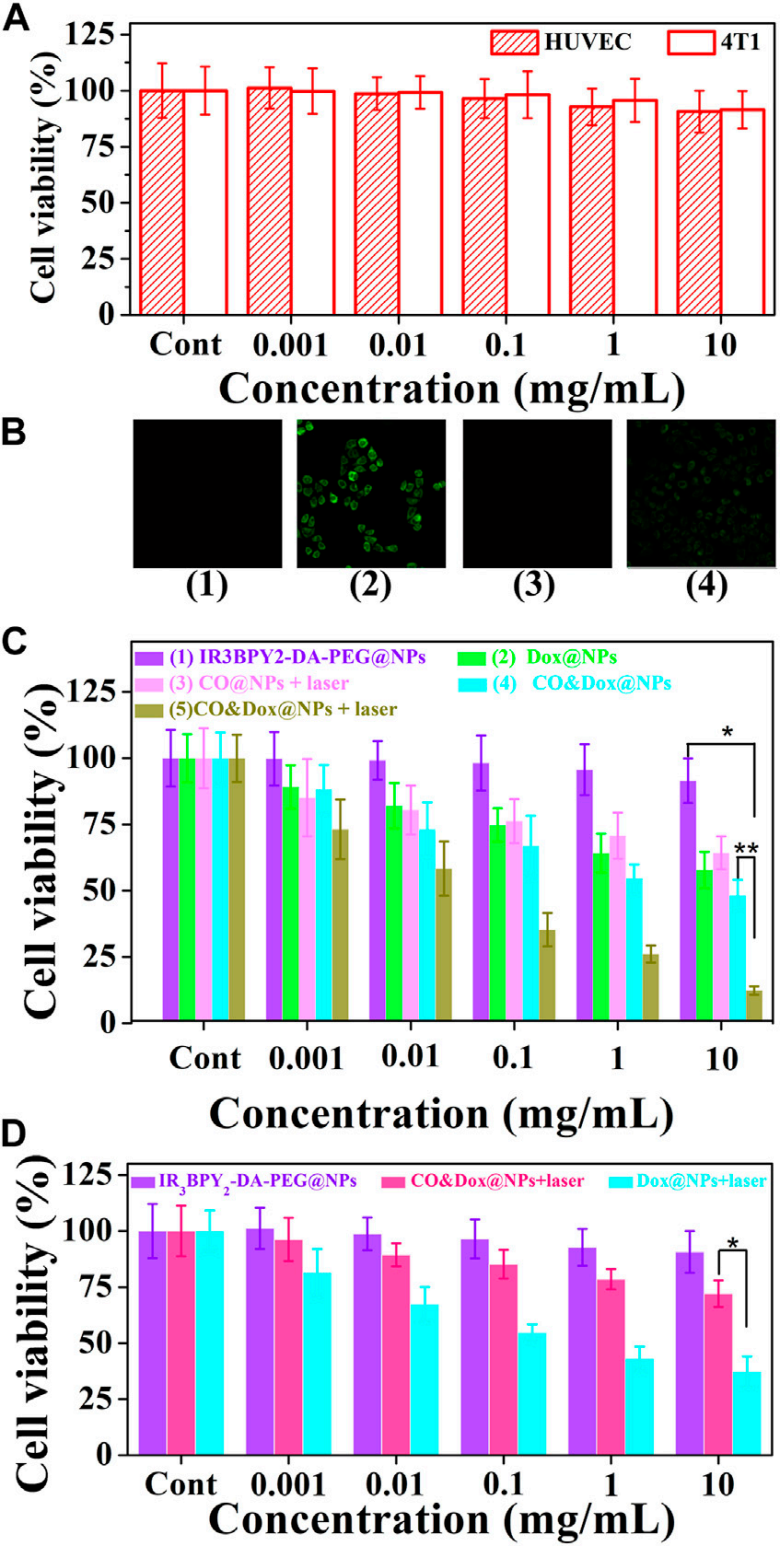
Cell experiment. **(A)** Cytotoxicity profiles of both HUVEC and 4T1 cells. **(B)** Intracellular controlled release of CO from IR_3_BPY_2_-DA-PEG@NPs with irradiation (1), CO&Dox@NPs with irradiation (2), CO&Dox@NPs without irradiation (3), and COPIRS with irradiation (4). **(C)** Cell viability of 4T1 after co-culturing with different samples and treatment: (1) IR_3_BPY_2_-DA-PEG@NPs, (2) Dox@NPs, (3) CO@NPs, (4) CO&Dox@NPs, and (5) CO&Dox@NPs. Groups 3 and 5 were subjected to NIR irradiation for 10 min. **(D)** Cell viability of HUVEC after co-culturing with IR_3_BPY_2_-DA-PEG@NPs, CO&Dox@NPs with irradiation, Dox@NPs with irradiation. The concentration is all in terms of IR_3_BPY_2_-DA-PEG@NPs, and “Cont” means the concentration is 0. *p < 0.001, **p < 0.05.

The safety of the nanocarrier (CO@NPs) should be evaluated prior to their use *in vivo*. The cytotoxicity of the CO@NPs at different concentrations without Dox and laser was evaluated through the MTT assay using HUVEC and 4T1 cells. These CO@NPs showed little toxicity to either normal cells or tumor cells (Figure 6A). Following co-culture, even at concentrations as high as 10 mg/ml, the cell survival rates were 90.7% and 91.5% compared to the control group, respectively, indicating that CO@NPs had a low cytotoxicity.

The intracellular generation of CO by these CO&Dox@NPs under NIR irradiation was also verified by a CO-detecting fluorescent probe: COP-1 (Michel et al., 2012) (Figure 6B). COP-1 itself did not exhibit fluorescence properties but it selectively reacted with CO to generate green fluorescence (λ_ex_ = 475 nm, λ_em_ = 507 nm). Clearly, green fluorescence was observed in the cells following co-culture with CO&Dox@NPs under NIR light irradiation (group 2), demonstrating the production of CO. However, a minimal amount of CO was detected in cells co-cultured with IR_3_BPY_2_-DA-PEG@NPs with laser (group 1) and CO&Dox@NPs without laser irradiation (group 3). Furthermore, CO was also detected when cells were co-cultured with COPIRS under NIR light irradiation, although the fluorescent intensity was lower than that of CO&Dox@NPs (group 4). These results indicate that the amount of COPIR entering cells is much lower than that of CO&Dox@NPs and that CO&Dox@NPs had enhanced ability to be endocytosed by tumor cells due to the FA modification on their surface (Supplementary Figure S2).

The *in vitro* anti-tumor efficacy of different samples against 4T1 cells was analyzed through the MTT assay. The IR_3_BPY_2_-DA-PEG@NPs without the encapsulation of CO and Dox showed no cytotoxicity without irradiation (groups 1) (Figure 6C). Dox@NPs, without the encapsulation of CO, showed obvious cytotoxicity because of the cytotoxicity of Dox (group 2). CO@NPs without the encapsulation of Dox and with irradiation showed some degree of cytotoxicity because of the cytotoxicity of CO and heat generated by COPIRS (group 3). CO&Dox@NPs showed a high cytotoxicity both with and without irradiation (group 5 and 4), because even without laser irradiation, -MnBr(CO)_3_ in COPIRS can spontaneously release a small amount of CO to enhance the anti-tumor efficacy of Dox. However, in group 5, the highest anti-tumor effect was achieved because all the CO in COPIRS was released after NIR irradiation, thus potentiating the anti-tumor effect of Dox. These results proved that CO has a chemotherapeutic sensitization effect on Dox and CO&Dox@NPs could be applied as an effective anti-tumor agent.

The proliferation of HUVEC cells co-cultured with different samples was also assessed by the MTT assay (Figure 6D). The IR_3_BPY_2_-DA-PEG@NPs had little impact on the proliferation of HUVEC cells, indicating that the IR_3_BPY_2_-DA-PEG@NPs itself showed little cytotoxicity to normal cells. However, the survival rate of HUVEC cells in the CO&Dox@NPs plus laser group was much higher than that of the Dox@NPs plus laser group, indicating that CO can inhibit the toxic effects of chemotherapeutic drugs (Dox) on normal cells. Thus, CO&Dox@NPs confer chemotherapeutic protection on normal cells and could increase the normal cell’s drug tolerance concentration about 1000-fold.

### *In Vivo* Anti-Tumor Effect

**FIGURE 7:**
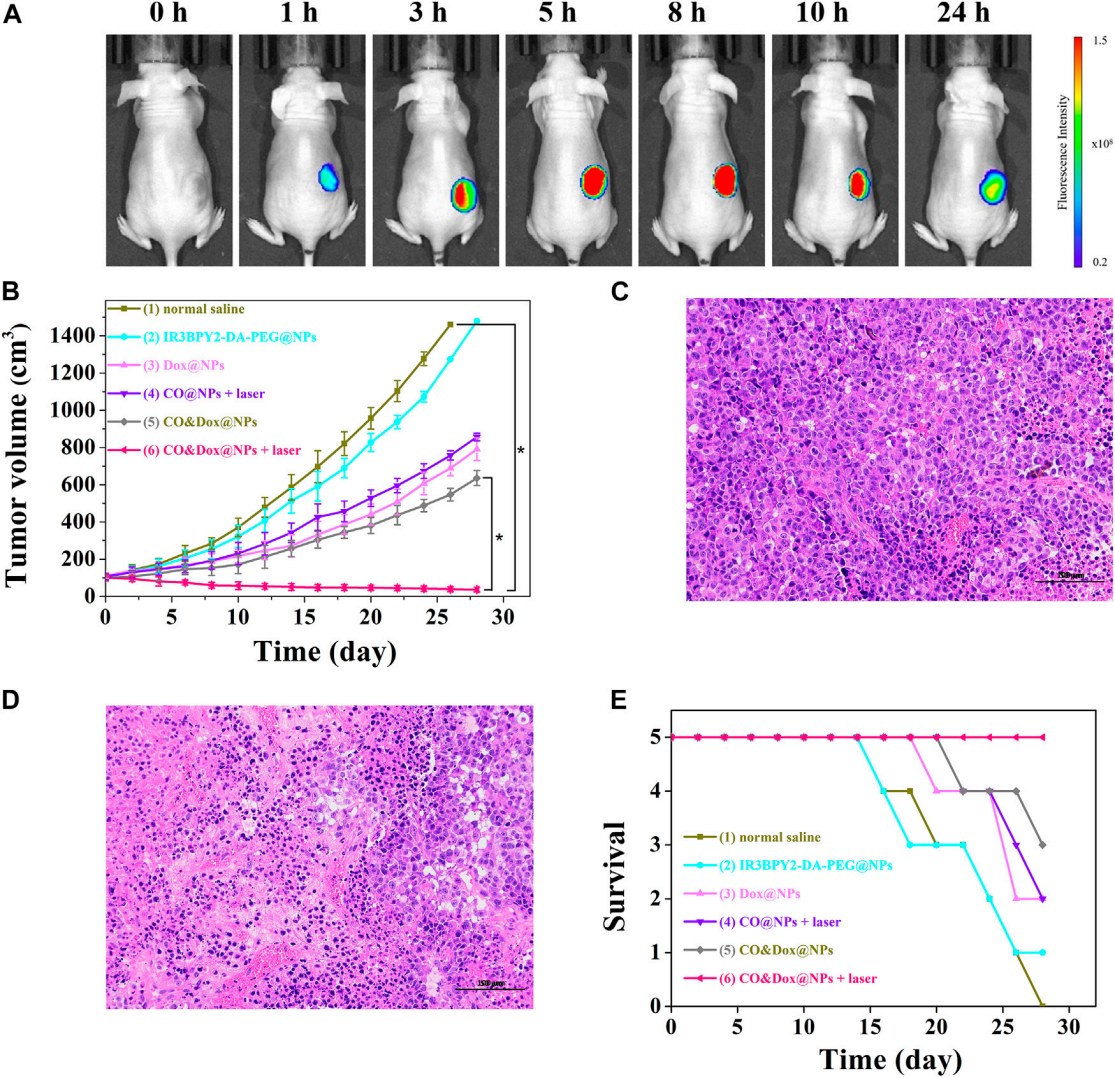
**(A)** *In vivo* fluorescence imaging of tumor bearing mouse before intravenous (i.v.) injection with CO&Dox@NPs and 1, 3, 5, 8, 10, and 24 h post i.v. injection with CO&Dox@NPs. **(B)** Tumor volumes of mice receiving different treatments; **(C)** Pathological section of tumor tissue from group (1) via H&E staining; **(D)** Pathological section of tumor tissue from group (6) via H&E staining. Scale bars: 50 μm; **(E)** Survival curves of mice in each group (*n* = 5 each group). Each mouse was intravenously injected with normal saline (100 μl) (1), IR_3_BPY_2_-DA-PEG@NPs (200 mg/kg) (2), Dox@NPs (200 mg/kg) (3), CO@NPs (200 mg/kg) (4), CO&Dox@NPs (220 mg/kg) (5), CO&Dox@NPs (220 mg/kg) (6), and groups 4 and 6 were treated with 808 nm laser for 10 min at 6 h post the incubation. *p < 0.001.

Nanoparticles with a size of 100–200 nm will accumulate in tumor tissue after intravenous (i.v.) injection because of the enhanced permeability and retention effect (Maeda et al., 2000; Kang et al., 2020). To further demonstrate the accumulation of nanomedicine in tumors, *in vivo* fluorescence imaging of tumor bearing mouse was evaluated. As shown in Figure 7A, CO&Dox@NPs exhibited good tumor targeting, and maximal accumulating concentration occurs between 5 and 8 h after i.v. injection. Therefore, we chose 6 h after injection as the time point of laser treatment.

Since CO&Dox@NPs showed an excellent anti-tumor effect *in vitro*, we speculated that they would also show remarkable anti-tumor effect *in vivo*. When the tumor size reached ∼100 mm^3^, the animals were randomly divided into six groups (*n* = 5) and treated differently. The tumor volume was measured and calculated every 2 days for 28 days (Figure 7B). As a control group, the tumor volume in mice injected with saline increased rapidly during the observation period (group 1) and the IR_3_BPY_2_-DA-PEG@NPs alone had little effect on tumor volume. Only CO or Dox treatment inhibited tumor growth to some extent but could not stop tumors from growing (groups 3 and 4). However, when treated with the synergistic delivery of CO and Dox (group 6), the tumor volume was severely suppressed and the tumors were almost gone at the end of treatment. Pathological section of tumor tissue further supported the above results.We can find that the tumor tissue of the mouse treated with saline (group 1) is very intact (Figure 7C). But in the tumor tissue of the mouse treated with the synergistic delivery of CO and Dox (group 6), the morphology of tumor cells has undergone great changes, and there are a large number of apoptotic tumor cells (Figure 7D). This demonstrated that CO delivered in coordination with Dox can greatly enhance the therapeutic effect of Dox on tumors, indicating that CO&Dox@NPs lead to chemotherapy sensitization.

The survival rates of mice with the different treatments are shown in Figure 7E. The death of mice first appeared on days 16 (normal saline), 16 (IR_3_BPY_2_-DA-PEG@NPs), 20 (Dox@NPs), 22 (CO@NPs + laser), 22 (CO&Dox@NPs), while no mouse died in the group treated with CO&Dox@NPs with laser irradiation until the end of the observation period (28 days). At end of the observation period, 0 (normal saline), 1 (IR_3_BPY_2_-DA-PEG@NPs), 2 (Dox@NPs), 2 (CO@NPs + laser), 3 (CO&Dox@NPs), and 5 (CO&Dox@NPs + laser) mice survived, respectively. These results indicate that CO&Dox@NPs plus laser irradiation showed the best anti-tumor effect.

### Long-Term Pathological Study

**FIGURE 8:**
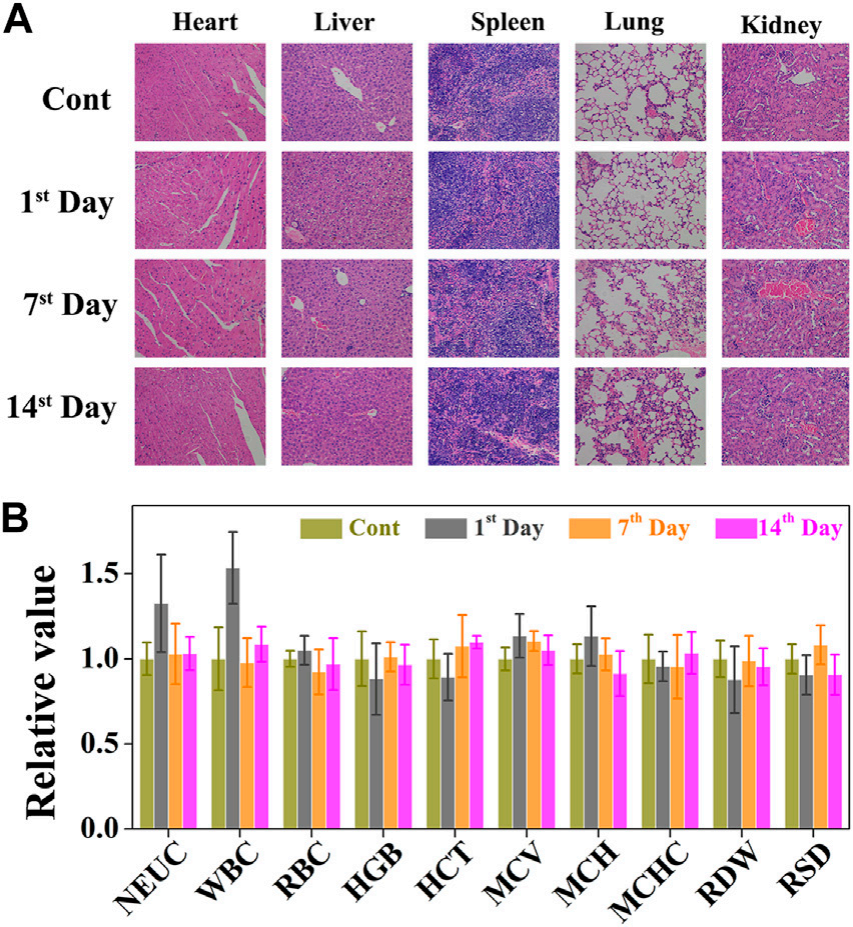
**(A)** The cytotoxic effect caused by CO&Dox@NPs with irradiation in major organs via H&E staining (heart, liver, spleen, lung, and kidney). **(B)** Serum biochemical study and hematology assay of neutrophilic granulocyte (NEU), white blood cell (WBC), red blood cells (RBC), hemoglobin (HGB), hematocrit (HCT), mean corpuscular volume (MCV), mean corpuscular hemoglobin concentration (MCH), mean corpuscular hemoglobin concentration (MCHC), coefficient variation of red cell distribution (RDW), and standard deviation in red cell distribution (RSD) levels at 1, 7, and 14 days.

Although CO&Dox@NPs showed promising tumor inhibition both *in vitro* and *in vivo*, their long-term *in vivo* safety should be further evaluated (Figure 8A). Six hours after the intravenous injection of CO&Dox@NPs following the 808 nm laser irradiation for 10 min, the tumor-bearing mice were sacrificed at predetermined time intervals (pre-treatment, days 1, 7, and 14 post treatment) and their five major organs (heart, liver, spleen, lung, and kidney) were resected. H&E staining test was performed to evaluate the pathological condition. No apparent pathological changes, such as inflammation lesions or abnormalities from tissue sections of mice, were observed on days 1, 7, and 14 post injection. The blood of tumor-bearing mice was also collected during the harvest of their organs for blood biochemical tests. The values of these biomarkers were close to the normal level on the first day post treatment, except for neutrophilic granulocytes and white blood cells, indicating an inflammatory response likely due to the therapeutic action during treatment (Figure 8B). Subsequently, the values returned to normal on days 7 and 14 post treatment. All these results preliminarily confirm the biocompatibility of CO&Dox@NPs *in vivo*, which ensures CO&Dox@NPs as an integrating CS and CP agent for safe and enhanced tumor chemotherapy.

## Conclusion

Herein, we demonstrated an effective strategy to enhance tumor chemotherapy by integrating CS and CP in the same nanomedicine. COPIRS was synthesized and showed an induction of the release of CO from carbonyl manganese and the generation of heat under NIR irradiation. Then, COPIRS was used as hydrophobic ends and PEG was introduced through DA group to form thermal-responsive amphiphilic copolymers. CO&Dox@NPs were prepared by self-assembly of COPIRS-DA-PEG encapsulating of Dox. Under NIR light irradiation, photoelectron generated from IR808 can induce carbon monoxide-releasing molecules to release CO, whereas heat could cause the breakdown of COPIRS-DA-PEG by the retro-Diels–Alder reaction. Internal heat then accelerates the collapse of nanomedicine, thus achieving the synergistic release of CO and Dox under a single NIR stimulus. The CO that is co-released with Dox could distinguish tumor cells and normal cells, and enhance the inhibition effect of Dox on tumor cells while, on normal cells, the inhibition effect of Dox was reduced. This strategy could not only enhance the chemotherapy effect but also reduce the toxic effect of chemotherapy drugs, thus improving the therapeutic effect of chemotherapy. Based on this, we hope that this treatment strategy can solve the current dilemma of chemotherapy and play an active role in clinical application.

## Data Availability

The original contributions presented in the study are included in the article/Supplementary Material, further inquiries can be directed to the corresponding authors.
