# Supplementary material for: Tumor Cell Distinguishable Nanomedicine Integrating Chemotherapeutic Sensitization and Protection
Source: Front Bioeng Biotechnol. 2021 Nov 8;9:773021. doi: 10.3389/fbioe.2021.773021 (PMC8631718; doi:10.3389/fbioe.2021.773021)
Supplement: Supplementary file 1 [file DataSheet1.docx]

Supplementary Material

# Supplementary Figures and Tables


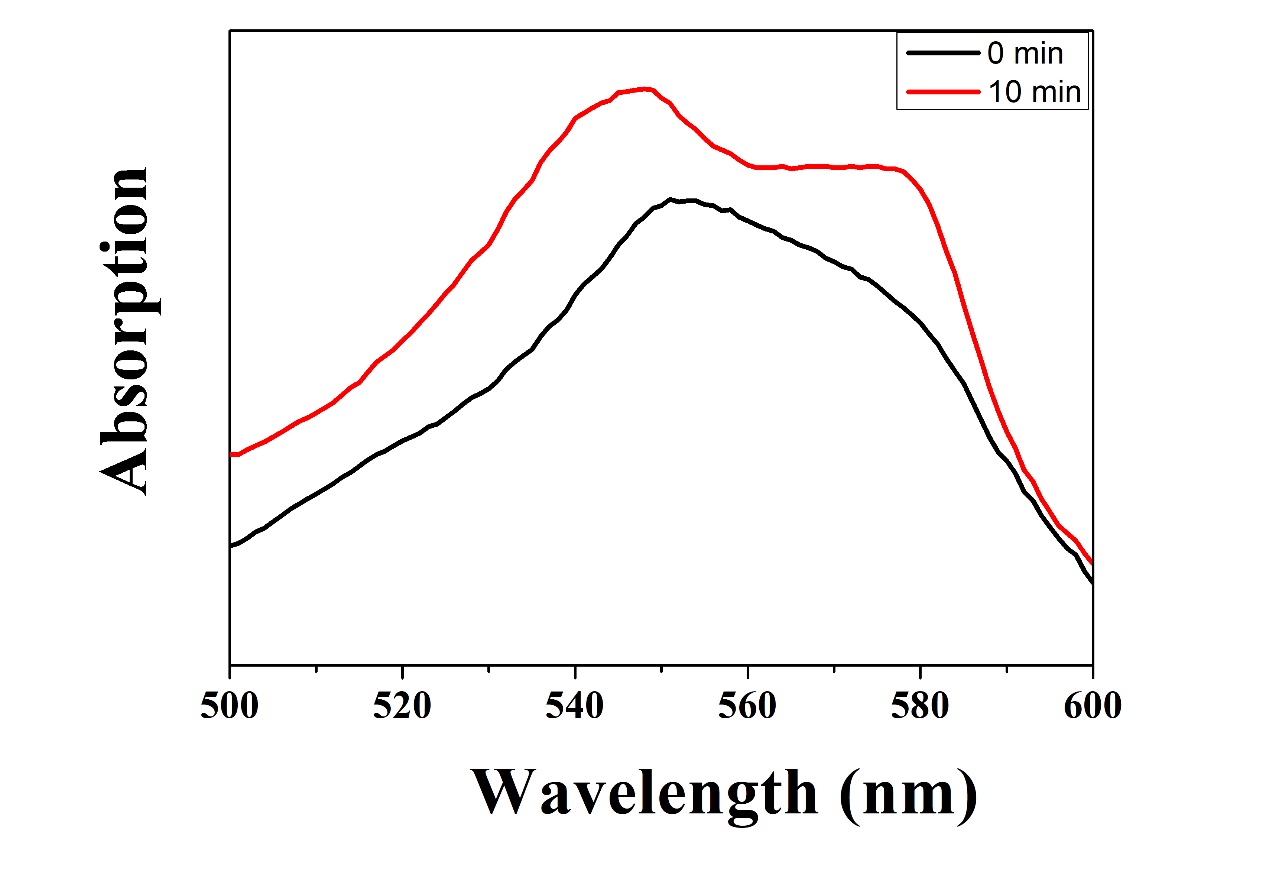


Fig S1 UV-vis spectra of deoxy-Mb solution co-incubated with COPIRS before NIR irradiation and at 10 min after NIR irradiation.


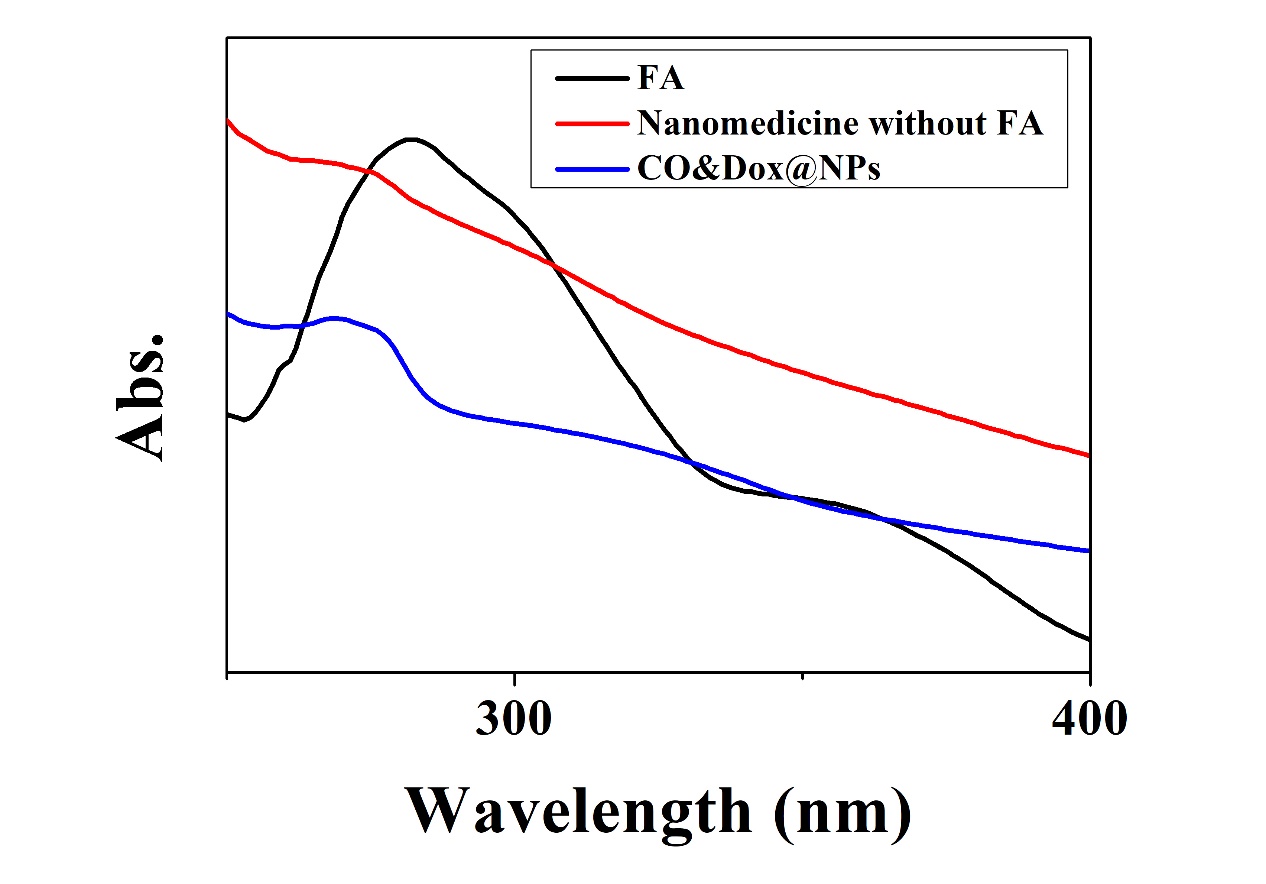


Fig S2 UV-vis absorption spectra.

“Nanomedicine without FA” were prepared in the same way as CO&Dox@NPs: 50 mg of PCL-DA-PEG, 5 mg of COPIRS, and 3 mg of Dox was dissolved in 5 mL of methylene chloride and then added to 50 mL of DI water. The mixture was emulsified by ultrasound for 10 min and then methylene chloride was removed through a rotary evaporator. As the absence of PCL-PEG-FA, “Nanomedicine without FA” does not contain FA. Then, the UV-vis absorption spectra of the solution of FA, Nanomedicine without FA, and CO&Dox@NPs were measured. As shown in Fig S1, absorption peak of FA at 282 nm could be observed. No absorption peak could be found in the absorption spectra of “Nanomedicine without FA”, but the absorption peak appeared in the absorption spectra of CO&Dox@NPs, confirming the presence of folic acid in CO&Dox@NPs.
